# Different distribution of breast ductal carcinoma *in situ*, ductal carcinoma *in situ* with microinvasion, and invasion breast cancer

**DOI:** 10.1186/1477-7819-10-262

**Published:** 2012-12-08

**Authors:** Zhang Wei, Gao Er-li, Zhou Yi-li, Zhai Qi, Zou Zhang-yong, Guo Gui-long, Chen Guo-rong, Zheng Hua-min, Huang Guan-li, Zhang Xiao-hua

**Affiliations:** 1 Department of Oncology, The First Affiliated Hospital of Wenzhou Medical College, Wenzhou, Zhejiang, People’s Republic of China; 2 Department of Pathology, The First Affiliated Hospital of Wenzhou Medical College, Wenzhou, Zhejiang, People’s Republic of China; 3 Department of Radiology, The First Affiliated Hospital of Wenzhou Medical College, Wenzhou, Zhejiang, People’s Republic of China

**Keywords:** Breast neoplasms, Ductal carcinoma *in situ*, Ductal carcinoma *in situ* with microinvasion, Invasion breast cancer

## Abstract

**Background:**

Breast ductal cancer *in situ* (DCIS) can recur or progress to invasive ductal cancer (IDC), and the interim stage include DCIS with microinvasion (DCIS-Mi). In this article, we attempt to study the study the differences of clinicopathological features, imaging data, and immunohistochemical-based subtypes among DCIS, DCIS-Mi, and IDC.

**Methods:**

In this retrospective study, we attempt to compare the clinicopathological features, immunohistochemical results and imaging data of 866 patients (included 73 DCIS, 72 DCIS-Mi, and 721 IDC).

**Results:**

Patients with DCIS and DCIS-Mi were younger than those with IDC (*P* = 0.007). DCIS and DCIS-Mi often happened in premenopausal women while IDC was opposite (*P* <0.001). The incidence of IDC with node-positive was significantly higher than it in DCIS and DCIS-Mi (*P* <0.001). We also observed that the Her2-positive was more often found in patients with pure DCIS compared to those with DCIS-Mi and DCIS-I (*P* <0.001). There was a significant difference between the four subgroups (Luminal-A, Luminal-B, ERBB2+, Basal-like) from DCIS, DCIS-Mi, and IDC (*P* <0.001). Basal-like patients were fewer than other subgroups in DCIS, DCIS-Mi, and IDC. The incidence of the first performance of ultrasound (catheter winded and nodular mass) and mammography (nodular mass) had significantly difference among patients with DCIS, DCIS-Mi, and IDC (*P* <0.001).

**Conclusions:**

Different clinicopathological, immunohistochemical, and imaging features among DCIS, DCIS-Mi, and IDC indicate that they are distinct entities. A larger sample size is needed for further study.

## Background

Breast cancer is one of the most common malignant tumors for women [1]. In recent years, the incidence of breast cancer shows an increasing trend in China. Breast ductal cancer *in situ* (DCIS) is a neoplastic proliferation of epithelial cells confined to the ductal-lobular system without tumor invasion through the basement membrane [2]. Due to the extensively use of mammographic imaging, the number of patients with DCIS and DCIS with microinvasive (DCIS-Mi) is increasing. According to the criteria of the American Joint Committee on Cancer (AJCC), DCIS-Mi is defined as DCIS with a microscopic focus of invasion ≤1 mm in the longest diameter, which accounts for approximately 10% to 20% of DCIS cases [3,4]. DCIS-Mi included the dominant lesion, which is *in-situ* carcinoma and one or more foci of infiltration [5-8]. And international scholars consider that it may be the interim stage in the progression from DCIS to invasive breast cancer (IDC) [9,10]. Recent studies revealed that DCIS-Mi was potential for invasion and metastasis differentiated form pure DCIS, which also result for the different surgical strategy [11,12]. So DCIS-Mi may represent a distinct entity.

Today a multistep model of human breast cancer progression hypothesizes that IDC develops through sequential stages, from premalignant hyperplastic breast lesions with or without atypia to carcinoma *in situ* to invasive carcinoma [2,13-15]. Very few studies have paid attention to the association of clinicopathological and immunohistochemical (IHC) features among DCIS, DCIS-Mi, and IDC. So we studied the differences of clinicopathological features as well as IHC marker-based subtypes and imaging data among DCIS, DCIS-Mi, and IDC.

## Methods

### Patients

We retrospectively collected 953 breast cancer patients from February 2006 to April 2010. Among the 953 patients, 96 cases were excluded because their first tumor lumpectomies were not in our hospital and there were no data of specimens. Finally, 73 patients with DCIS (9.29%), 72 patients with DCIS-Mi (9.16%), and 721 patients with IDC (83.26%) were enrolled in this research. All the patients accepted physical examination, chest radioscopy, mammography, ECG, complete blood count, routine biochemical tests, and ultrasound (US) (included breasts, axillary fossa, abdomen, and pelvis). Each patient was treated with surgery by either lumpectomy or mastectomy, and whether doing axillary lymph node dissection was followed by adjuvant therapies according to the standard guideline. All patients were treated with lumpectomy or mastectomy (most with axillary lymph node dissection) followed by adjuvant therapies according to the standard guideline.

The age of the 866 patients ranged from 24 to 87 years old (the median age at diagnosis was 52 years old). Median follow-up of those patients was 35 months (range, 1 to 118 months). We collected age, menopausal status, tumor size, lymph node status, status of IHC (ER, PR, and HER2), family history, and the history of breast surgery. The pathologic and IHC outcomes were checked by our senior pathologists in our hospital. This study was approved by the Institutional Review Board of Wenzhou Medicine University. Written informed consents were obtained from the patients for publication of this report.

### Pathologic definition

DCIS is defined as clonal proliferation of cells growing within the basement membrane-bound structures of the breast [16]. DCIS-Mi is defined as DCIS with extension of cancer cells beyond the basement membrane and invasion ≤1 mm in the longest diameter. Those tumor cells which are invasive breast ductal basement membrane-bound structure were identified as IDC. The specimens of all patients in our study were diagnosed by three board-certified pathologists with a mean 11 years (17, 9, and 7 years, respectively) of experience who were not aware of the result of pathological examination from the former pathologist.

### Immunohistochemistry and scoring

The status of ER, PR, and HER2 confirmed by IHC staining, which was performed through a standard operating procedure in our Department of Pathology. ER and PR expression in the nuclei of tumor cells, Her-2 expression in the cytoplasm, positive cell is that cells contain brown grains. A score of 0 required no staining seen, 1 required ≤25 % of cells positive, 2 required 25% to 50% of cells stained, 3 required 50% to 75% of positive cells, and 4 required >75% of staining cells.

### Statistical analysis

We compared clinicopathological, IHC, and imaging characteristics in the patient with DCIS, DCIS-Mi, and IDC. For each group we estimated from the date of the surgery by the life table. Tests of association and correlation were conducted by using the n × n Pearson’s χ2 test (or Fisher’s exact test when appropriate). One-way ANOVA was used to compare continuous variables among two or more groups. *P* <0.05 was considered statistically significant. Statistical analyses were performed using SPSS software version 17.0.

## Results

The clinicopathological characteristics of patients are shown in Table 1. Patients with DCIS and DCIS-Mi were significantly younger than those with IDC (*P* = 0.007). Patients with DCIS and DCIS-Mi were more often found to have the status of premenopausal when compare to those with IDC (*P* ≤0.001). The incidence of lymph node metastasis from patients with IDC (38.75%) was significantly higher compared to the patients with pure DCIS (6.85%) and patients with DCIS-Mi (20.83%) (*P* ≤0.001). We also observed that the number of status of Her2 expressed positive in patients with DCIS and DCIS-Mi was higher than in those with IDC (*P* <0.001) but we found that the tumor size and family history of breast cancer in a first-degree relative seemed to have no difference in the three groups (*P* >0.05). We divided patients into four subgroups: Luminal-A, Luminal-B, ERBB2+, and Basal-like. We found no significant difference between the four subgroups regarding DCIS, DCIS-Mi, and IDC.

**Table 1:** Characteristics of the three patient populations: DCIS, DCIS-Mi, and IDC

**Parameter**	**DCIS**	**DCIS**-**Mi**	**IDC**	***P *value**
Total number	73	72	721	
Age (mean ± SD) (year)	48.96 ± 1.13	49.34 ± 1.01	52.23 ± 0.43	0.007
*Menopausal*				<0.001
Postmenopausal	21 (28.77)	28 (38.89)	415 (57.60)	
Premenopausal	52 (71.23)	44 (61.11)	306 (42.40)	
*Family history*				0.791
Positive	6 (8.2)	6 (8.3)	48 (6.7)	
Negative	67 (91.8)	66 (91.7)	673 (93.3)	
*Tumor size (cm)*				0.238
<2	38 (52.05)	32 (44.44)	302 (41.9)	
≥2	35 (47.95)	40 (55.56)	419 (58.1)	
*Lymph node*				0.000
Positive	5 (6.85)	15 (20.83)	279 (38.75)	
Negative	68 (93.15)	57 (79.17)	442 (61.25)	
*ER*				0.265
Positive	44 (60.27)	42 (58.33)	478 (66.25)	
Negative	29 (39.73)	30 (41.67)	243 (33.75)	
*PR*				0.736
Positive	41 (56.20)	37 (51.40)	405 (56.25)	
Negative	32 (43.80)	35 (48.6)	316 (43.75)	
*Her-2*				0.000
Positive	47 (64.38)	48 (66.67)	307 (42.60)	
Negative	26 (35.62)	24 (33.33)	414 (57.40)	
*Over Her-2*				0.000
Positive	35 (47.95)	36 (50.00)	180 (25.00)	
Negative	38 (52.05)	36 (50.00)	541 (75.00)	
*Subtype*				0.000
Luminal-A	23 (31.51)	21 (29.17)	325 (45.00)	
Luminal-B	26 (35.62)	25 (34.72)	162 (22.50)	
ERBB2+	21 (28.77)	23 (31.94)	144 (20.00)	
Basal-like	3 (4.10)	3 (4.17)	90 (12.50)	

*DCIS* Ductal carcinoma in situ; Family history, Family history of breast cancer in a first-degree relative; *IDC* Infiltrating ductal carcinoma; *Mi* Microinvasion; *SD* Standard deviation.

Table 2 showed the clinical data of the first clinical performances and diagnostic methods among DCIS, DCIS-Mi, and IDC groups. For the first clinical performance for diagnosis, palpable mass was the main symptom with no significant difference in the three groups (*P* = 0.52). The incidence of nipple discharge was similar in the patients with DCIS, DCIS-Mi, and IDC groups (*P* = 0.51), but DCIS and DCIS-Mi was mainly yellow discharge, IDC was mainly bloody. The second main clinical performance was asymptomatic. Patients often see doctors because of physical examination or abnormalities in imaging (included US and mammography). There was no difference between DCIS, DCIS-Mi, and IDC groups in eczema and pain for the first clinical performances (*P* < 0.05). For diagnostic methods, the proportion of catheter winded was less in IDC than in DCIS and DCIS-Mi (*P* ≤0.001), while the proportion of the nodular mass was significantly more in IDC than in DCIS and DCIS-Mi by US (*P* = 0.003). There was no difference between DCIS, DCIS-Mi, and IDC in solid and cystic, structural disorder, and calcification, respectively (*P* <0.05). For mammography feature, there was significantly difference among them in the imaging of nodular mass (*P* <0.05), but no difference in the imaging of calcification (*P* =0.431).

**Table 2:** **First clinical performances and diagnostic methods of the three patient populations**: **DCIS**, **DCIS**-**Mi**, **and IDC**

**Parameter**	**DCIS**	**DCIS**-**Mi**	**IDC**	***P *value**
Total number	73	72	721	
*First clinical performance*				
Nipple discharge	9 (12.3)	8 (11.1)	63 (8.7)	0.51
Bloody	4 (5.5)	3 (4.2)	44 (6.1)	0.776
Yellow	5 (6.8)	5 (6.9)	19 (3.3)	0.062
Palpable tumor	48 (65.8)	50 (69.4)	518 (71.8)	0.52
Pain	3 (4.1)	5 (5.9)	37 (5.1)	0.731
Eczema	1 (1.4)	1 (1.4)	29 (4.0)	0.22
Asymptomatic	12 (16.4)	8 (11.1)	74 (10.3)	0.27
*Diagnostic methods*				
Ultrasound				
Catheter widened	65 (89)	56 (77.8)	450 (62.4)	0.000
Solid and cystic	3 (4.1)	1 (1.4)	21 (2.9)	0.589
Nodular mass	56 (76.7)	53 (73.6)	648 (89.9)	0.003
Structural disorder	56 (76.7)	44 (61.6)	486 (67.4)	0.125
Calcifications	37 (50.7)	34 (47.2)	342 (47.7)	0.866
Mammography				
Calcifications	60 (82.2)	63 (87.5)	631 (87.5)	0.431
Nodular mass	30 (41.1)	25 (34.7)	532 (73.8)	0.000

*DCIS* Ductal carcinoma *in situ*; *IDC* Infiltrating ductal carcinoma; *Mi* Microinvasion.

A total of 215 of those 866 patients had a history of breast operation and biopsy: 19 (26.0%) DCIS, 20 (27.8%) DCIS-Mi, and 176 (24.4%) IDC. We divided the past disease into benign breast disease (BBD) and malignant breast disease (MBD) which were showed in Table 3*.* BBD is usually subdivided into non-proliferative lesions (fibrosis, cysts, apocrine metaplasia, fibroadenoma), proliferative lesions without atypia (lobular hyperplasia without atypia, sclerosing adenosis, papilloma), and hyperplasia with atypia (atypical ductal hyperplasia, atypical lobular hyperplasia). MBD includes IDC, invasive lobular carcinoma, invasive mucinous carcinoma, invasive tubular carcinoma, and DCIS. Then we found there was no difference between them in the past different breast benign disease types. And it was worth noting that lobular hyperplasia without atypical accounts for most. At the same time, one case of DCIS has gotten IDC in the past breast surgical or biopsy history.

**Table 3:** **Past breast surgical or biopsy history of the three patient populations**: **DCIS**, **DCIS**-**Mi**, **and IDC**

**Parameter**	**DCIS**	**DCIS**-**Mi**	**IDC**	***P *value**
Total number	73	72	721	
*Past breast surgical or biopsy history*	19 (26.0)	20 (27.8)	176 (24.4)	0.795
Benign				
Fibrosis	2 (10.5)	4 (20)	29 (16.5)	0.700
Cysts	3 (15.8)	2 (10)	23 (13.1)	0.864
Apocrine metaplasia	1 (5.3)	1 (5.0)	5 (2.8)	0.789
Fibroadenoma	1 (5.3)	1 (5.0)	6 (3.4)	0.885
Lobular hyperplasia without atypia	8 (42.1)	6 (30.0)	64 (37.2)	0.728
Sclerosing adenosis	0 (0)	0 (0)	1 (0.5)	
Papilloma	1 (5.3)	2 (10.0)	9 (5.2)	0.724
Atypical ductal hyperplasia	1 (5.3)	2 (10.0)	10 (5.8)	0.780
Atypical lobular hyperplasia	3 (15.8)	2 (10.0)	28 (16.3)	0.743
Malignant				
IDC				
Invasive lobular carcinoma	0	0	0	
Invasive mucinous carcinoma	0	0	0	
Invasive tubular carcinoma	0	0	0	
DCIS	0	0	1	

*DCIS* Ductal carcinoma in situ; *IDC* Infiltrating ductal carcinoma; *Mi* Microinvasion.

## Discussion

Breast cancer is the second leading cause of death due to cancer in women. In recent years, with the improvement of diagnostic methods, the detection rate of breast cancer has been increasing. DCIS accounts for approximately 10% to 15% of breast cancers detected by mammography in China [17,18]. Now the data suggest that carcinoma *in situ* can recur or progress to IDC and breast DCIS-Mi is considered to be the interim stage in the progression from DCIS to IDC [9,10]. The diagnosis and the ability to predict the outcome of patients with DCIS/DCIS-Mi are not satisfactory, leaded to inappropriate treatment choices. To the authors’ knowledge, the hunt for molecular prognostic markers for DCIS and DCIS-Mi has not succeeded. Therefore, we urgently want to find the difference in clinicopathological, IHC, and imaging features between DCIS, DCIS-Mi, and IDC in order to describe the process of DCIS to IDC from the clinical aspects and discuss those results, contact treatment, and reduce the incidence.

We studied the differences of clinicopathological, IHC, and imaging features among DCIS, DCIS-Mi, and IDC. We found that there was no significant difference between patients with DCIS, DCIS-Mi, and IDC in the first symptoms in patients with previous breast history and tumor size, but there were significant different in patients’ age and menopausal state. It suggested that even if patient get diseases the situations are consistent, patients with old age and menopause were more likely to have IDC. It was found that there was a significant difference in the number of patients with lymph node metastasis, and the former two were significantly less than the latter, suggesting that the latter degree of malignancy was significantly higher than former two. The first clinical performance, family history, and previous history of breast disease had no difference between DCIS, DCIS-Mi, and IDC, while the degree of malignancy in IDC was higher than in DCIS and DCIS-Mi. Except for the age and the status of menopause, we found that US and mammography played an important role in differentiating DCIS, DCIS-Mi, and IDC. US was usually routinely performed for patients because of its advantages (non-traumatic, repetitive), and it could find a mass which is >2 cm [19]. This paper studied the difference of US features among DCIS, DCIS-Mi, and IDC, and found that the mass imaging of IDC patients by US were more obvious, which may be due to IDC patients’ late incidence, large mass, type of intraductal carcinoma, calcification, and ill-defined infiltration. The number of DCIS and DCIS-Mi patients with catheter widened imaging by US was greater than IDC patients, which may be explained that the latter’s large tumor image cover the widened catheter. Mammography played an important role in the early detection of breast cancer, especially in DCIS. According to the literature, mammography was important for detecting calcifications with high sensitivity, especially for the detection accuracy rate of DCIS. We also studied the differences among them in the mammography imaging, and found that there was no significant difference in calcification, but significant differences found in mass images. This may be due to DCIS patients’ early incidence and large mass, and IDC, DCIS, and DCIS-Mi all had calcification, so mammography was not obvious, but important in the diagnosis of early DCIS with micro-calcification.

In molecular biology, the breast is a sex hormone-dependent organ. Its growth, development, and cell proliferation are all influenced by estrogen and progesterone. The regulation works by estrogen receptor (ER) and progesterone receptor (PR) binding the receptors in the breast cell. ER and PR play an important role in the incidence of breast cancer; normal breast tissue of ER-positive expression will increase the risk of breast cancer [20]. ER and PR were closely related to the prognosis and endocrine therapy. The positive expression of endocrine therapy is an effective rate of 80% ER-positive tamoxifen treatment which can effectively reduce the local recurrence of DCIS [21]. A report showed that ER and PR expression range from 60% to 78%in DCIS [22]. This article basically confirmed the literature. At the same time, we found molecular markers ER and PR expression were similar in DCIS, DCIS-Mi, and IDC, which suggested hormone receptor status was determined in DCIS stage. Although there was no accurate conclusion in the breast cancer pathway, it may be a very close relationship between DCIS and IDC in the event of the development and occurrence.

Her-2 proto-oncogene product is one of the epidermal growth factor receptor (EGFR) family, with binding with ligand, changing the conformation, and causing a series of ‘waterfall’ types of chain reaction, then accelerating cell proliferation, accelerating cell cycle, and enhancing malignant behavior. Most studies suggested that Her-2 over-expression was related to breast cancer invasion and poor prognosis [23,24]. Our study found that Her-2 positive expression was 64.3% in DCIS, 66.67% in DCIS-Mi, and 42.5% in IDC. Consistent with previous reports, Her-2 express was lower in IDC than in DCIS, and was over-expressed in DCIS [25-27]. At the same time, several studies have found that normal breast tissue or benign lesions generally do not over-express HER-2 [25,27]. Some researches thought Her-2 positive expression was due to the process of atypical hyperplasia in DCIS, but in developing into IDC Her-2 frequently lose, or Her-2 caused the direct immune response. Another assumption was that the Her-2 negative in IDC was not developed from DCIS, but from the atypical hyperplasia [28,29]. According to data, we agreed with this hypothesis. So we regarded Her-2 as one of the indicators of prognosis and treatment.

We then tried to discover whether patients with DCIS, DCIS-Mi, and IDC were associated with a history of breast disease. Now due to the development of breast imaging examination, lesions could be found in the early stages. Breast disease history is helpful to evaluate the subsequent risk of breast cancer. Previous reports showed that women with proliferative breast lesions without atypia have a slightly increased risk of breast cancer, whereas women with atypical hyperplasia have a substantially increased risk [13,30-34]. In this retrospective study of breast operation history or biopsy history of patients who got DCIS, DCIS-Mi, and IDC, we found there was no difference in the proportion of previous breast operation and biopsy. We also found that DCIS, DCIS-Mi, and IDC patients whose previous medical history of lobular hyperplasia without atypia accounts for the large proportion may be explained by its high incidence in normal people. At the same time, hyperplasia with atypia accounts less than lobular hyperplasia without atypia, but it was accounted more than other breast disease and it also agreed with the above point of view. Finally, one case of DCIS patients recurred into IDC, indicating that DCIS has the possibility of recurrence. However, this retrospective study excluded patients with breast cancer who had not performed biopsy or operation, so it was not very accurate, and further analysis remains to be systematic prospective studies and large-scale system examination.

## Conclusions

In summary, through comparing DCIS, DCIS-Mi, and IDC, we found that DCIS, DCIS-Mi, and IDC were distinct entities. A larger sample size is needed for further study.

## Abbreviations

BBD: Benign breast disease; DCIS: Breast ductal cancer *in situ*; DCIS-Mi: Breast ductal cancer *in situ* with microinvasion; IDC: Invasive ductal cancer; IHC: Immunohistochemical; MBD: Malignant breast disease; US: Ultrasound.

## Competing interests

There are no financial or non-financial competing interests in our study.

## Authors’ contributions

ZW and GG-L drafted the manuscript. GE-L and ZY-L participated in the design of the study and performed the statistical analysis. ZQ and ZZ-Y conceived of the study, and participated in its design and coordination, and helped to draft the manuscript. CG-R is the main pathologist for diagnose the specimens and ZH-M is main radiologist for evaluating the imaging of Ultrasound and Mammography. HG-L and ZX-H made up the surgical team involved in the most of patients. All authors read and approved the final manuscript.

## Authors’ information

Zhang Wei is a graduate of Wenzhou Medical College, Wenzhou, Zhejiang, People’s Republic of China. Gao Er-Li is a graduate of Wenzhou Medical College, Wenzhou, Zhejiang, People’s Republic of China. Zhou Yi-Li is a graduate of Wenzhou Medical College, Wenzhou, Zhejiang, People’s Republic of China. Zhai Qi is a graduate of Wenzhou Medical College, Wenzhou, Zhejiang, People’s Republic of China. Zou Zhang-Yong is a graduate of Wenzhou Medical College, Wenzhou, Zhejiang, People’s Republic of China. Guo Gui-Long is Chief Physician of Department of Oncology, The First Affiliated Hospital of Wenzhou Medical College, Wenzhou, Zhejiang, People’s Republic of China. Chen Guo-Rong is Chief Physician of Department of Pathology, The First Affiliated Hospital of Wenzhou Medical College, Wenzhou, Zhejiang, People’s Republic of China. Zheng Hua-Min is a radiologist of Department of Radiology, The First Affiliated Hospital of Wenzhou Medical College, Wenzhou, Zhejiang, People’s Republic of China. Huang Guan-li is a surgeon of department of Oncology, The First Affiliated Hospital of Wenzhou Medical College, Wenzhou, Zhejiang, People’s Republic of China. Zhang Xiao-hua is Chief Physician of department of Oncology, The First Affiliated Hospital of Wenzhou Medical College, Wenzhou, Zhejiang, People’s Republic of China.
